# Consumption of Added Sugars by States and Factors Associated with Added Sugars Intake among US Adults in 50 States and the District of Columbia—2010 and 2015

**DOI:** 10.3390/nu15020357

**Published:** 2023-01-11

**Authors:** Seung Hee Lee, Sohyun Park, Heidi M. Blanck

**Affiliations:** Division of Nutrition, Physical Activity, and Obesity (DNPAO), National Center for Chronic Disease Prevention and Health Promotion (NCCDPHP), Centers for Disease Control and Prevention (CDC), 4770 Buford Highway, NE, Atlanta, GA 30341, USA; geo7@cdc.gov (S.P.); hcb3@cdc.gov (H.M.B.)

**Keywords:** added sugars, adults, nutrition, dietary intake, National Health Interview Survey

## Abstract

Purpose: The high intake of added sugars from foods or beverages increases the risk of obesity, hypertension, dyslipidemia, and cardiovascular disease. Because state-level data are lacking, we estimated dietary intake of added sugars by state and factors associated with intake among US adults. Design: Nationally representative, cross-sectional, in-person, household survey. Setting: 50 states and DC. Sample: 52,279 US adults from pooled data from 2010 and 2015 National Health Interview Surveys. Measures: Estimated total added sugars intake (tsp/day) using the National Cancer Institute’s scoring algorithm that converts responses from the Dietary Survey Questionnaire screener to estimated total added sugars intake (tsp/day). Analysis: Mean dietary-added sugars intake estimates and standard error were calculated for adults’ characteristics and by state for all 50 states and the District of Columbia. Differences by adult’s characteristics were assessed by pairwise *t*-tests (*p* < 0.05). All analyses accounted for complex survey design and sampling weights. Results: Overall, US adults consumed 17.0 tsp of added sugars/day (range: 14.8 tsp/day in Alaska to 21.2 tsp/day in Kentucky). Added sugars intake varied by states and sociodemographic characteristics. Conclusion: Findings may inform efforts to reduce added sugars intake to lower the high burden of chronic disease.

## 1. Introduction

Added sugars intake provides additional calories with poor nutritional value and increases the risk of chronic diseases, including obesity [1], hypertension [2], dyslipidemia [3], and cardiovascular disease mortality [4]. The Dietary Guidelines for Americans (DGA) state that added sugars should account for less than 10% of daily calories [5]. Based on the 2015–2016 National Health and Nutrition Examination Survey, 12.7% of total daily calories were from added sugars among US adults [6]. There are no state-specific data on added sugars intake among adults. Having state-specific data can inform states’ various intervention strategies and programs on added sugars intake. Therefore, we estimated dietary intake of added sugars by 50 states and DC and examined factors associated with intake among US adults.

## 2. Methods

The National Health Interview Survey (NHIS) is a nationally representative, cross-sectional, in-person, household survey conducted by the National Center for Health Statistics (NCHS) [7]. One sample adult is randomly selected from each family in the household to complete more detailed questions about their health, including The Cancer Control Supplement (CCS), which contains the National Cancer Institute (NCI) Dietary Screener Questionnaire (DSQ). The CCS was administered both in 2010 and in 2015 to assess individuals’ dietary intakes and was approved by the NCHS Research Ethics Review Board. We used nationally weighted data from combined 2010 [8] and 2015 [9] NHIS CCS to examine the state-specific estimated dietary intake of added sugars (teaspoons (tsp)/day) in 50 states and DC and by sociodemographic characteristics. Data were combined for more stable estimates in obtaining state-specific findings. We used nationally weighted data from combined 2010 and 2015 NHIS CCS to examine the prevalence of added sugars intake among 52,279 US adults aged 18 or older. This study required the use of restricted NHIS files for state estimates and categorizing metropolitan status available through the NCHS Research Data Center.

Estimated dietary-added sugars intake was calculated based on respondents’ answers to 9 questions: During the past month, how often did you (1) “… drink regular soda or pop that contains sugars? Do not include diet soda.”; (2) “… drink SPORTS and ENERGY drinks such as Gatorade, Red Bull, and Vitamin water?”; (3) “… drink sweetened fruit drinks, such as Kool-Aid, cranberry, and lemonade? Include fruit drinks you made at home and added sugars to.”; (4) “… drink coffee or tea that had sugars or honey added to it? Include coffee and tea you sweetened yourself and presweetened tea and coffee drinks such as Arizona Iced Tea and Frappuccino. Do not include artificially sweetened coffee or diet tea.”; (5) “… eat chocolate or any other types of candy? Do not include sugars-free candy.”; (6) “… eat doughnuts, sweet rolls, Danishes, muffins, or pop-tarts? Do not include sugars-free items.”; (7) “… eat cookies, cake, pie, or brownies? Do not include sugars-free kinds.”; (8) “… eat ice cream or other frozen desserts? Do not include sugars-free kinds.”; (9) “… eat hot or cold cereals?” [10].

The responses were collected by times per day, per week, or per month. The NCI’s scoring algorithm converts screener responses to estimated total added sugars sintake (tsp/day, where 1 tsp = 4 g) [11]. The explanatory variables were age (18–24, 25–39, 40–59, ≥60 years), sex (male, female), race/ethnicity (non-Hispanic White, non-Hispanic Black, Hispanic, non-Hispanic other), marital status (married/domestic partnership, not married), education (<high school, high school/GED, some college, college graduate), annual household income (<$35,000, $35,000–$74,999, $75,000–$99,999, ≥$100,000), metropolitan/nonmetropolitan status (metropolitan, nonmetropolitan) [12], and census region (Northeast, Midwest, South, West) [13]. Mean dietary-added sugars intake (tsp/day) estimates and standard error were calculated for adults’ characteristics and by state for all 50 states and the District of Columbia. Differences by respondent characteristics were assessed by pairwise *t*-tests (*p* < 0.05). All analyses were performed using SAS-Callable SUDAAN version 9.0 (RTI), accounting for complex survey design and sampling weights.

## 3. Results

Overall, the estimated mean dietary intake of added sugars was 17.0 tsp/day among US adults in 2010 and 2015. We found a significantly higher dietary intake of added sugars among adults younger than 60 years (range: 16.3–21.3 tsp/day) compared to adults aged ≥60 years (13.5 tsp/day) and males (19.7 tsp/day) compared to females (14.4 tsp/day). The estimated mean dietary intake of added sugars was significantly higher among non-Hispanic Black adults (17.9 tsp/day) and Hispanic adults (17.9 tsp/day) but significantly lower among non-Hispanic other adults (15.5 tsp/day) compared to non-Hispanic White adults (16.8 tsp/day). Adults who were not married had significantly higher dietary intakes of added sugars (17.5 tsp/day) compared to adults who were married or in domestic partnerships (16.7 tsp/day). Adults with lower education level (<high school, 18.9 tsp/day; high school/GED, 18.4 tsp/day; and some college, 17.1 tsp/day) had significantly higher mean dietary-added sugars than the college graduates (14.9 tsp/day). By annual household income level, estimated mean dietary-added sugars was significantly higher among those with incomes of <$35,000 (18.0 tsp/day), $35,000–$74,999 (17.2 tsp/day), and $75,000–$99,999 (16.7 tsp/day) compared to those with incomes of ≥$100,000 (15.6 tsp/day). Nonmetropolitan residents had significantly higher mean dietary intakes of added sugars (18.5 tsp/day) compared to metropolitan residents (16.8 tsp/day). By census region, those residing in the South (17.8 tsp/day), the Midwest (17.3 tsp/day), and the Northeast (16.5 tsp/day) had significantly higher mean dietary intakes of added sugars than those residing in the West (16.0 tsp/day) (Table 1). By states, estimated mean dietary intake of added sugars ranged from 14.8 tsp/day in Alaska to 21.2 tsp/day in Kentucky (Table 2).

## 4. Discussion

US adults consumed about 17.0 tsp/day of dietary-added sugars in 2010 and 2015, which is similar to findings from 2015 to 2016 that showed on average 16.2 tsp equivalent of added sugars intake among Americans (≥20 years) on a given day [14]. These intakes are at a higher level of added sugars than suggested by major groups, including the American Heart Association, which suggests that most men and women should consume no more than 9 and 6 tsp/day of added sugars, respectively [15]. In 2015–2016, only 47% of US adults met the 2015–2020 DGA recommendation for added sugars (accounting for less than 10% of daily total calories) [6].

Added sugars intake varied by states (range: 14.8 tsp/day in Alaska to 21.2 tsp/day in Kentucky) and sociodemographic characteristics, which may contribute to chronic disease disparities. Other studies have also found differences in consumption by geography [16]. In 2014, the prevalence of daily SSB intake was higher among US adults living in Midwest (70.2%) and South (69.4%) regions compared to those living in Northeast (66.3%) and West (66.3%) regions [16], which is similar to our study that showed the West with the lowest and the South with the highest mean added sugars intakes.

Our study has several limitations. The data were collected using an FFQ and do not necessarily represent all consumption. The data were collected between 2010 and 2015 and may not reflect current consumption, particularly because evidence from other research shows that added sugars consumption has declined from 85 g (21.25 tsp) in 2003–2004 to 72 g (18 tsp) in 2017–2018 [17]. In addition, combining data could mask changes that occurred during the study period. However, even though data are older, this is the only study to our knowledge to examine added sugars intake for all 50 states and the District of Columbia by using a nationally representative sample of US adults. Third, sugars intake is expressed as weight (g), not percent energy. Undertaking and presenting the analyses as % energy would have been a useful addition or alternative. If there were between-state differences in reported total energy intake, the differences in sugars intakes could in part be explained. However, NHIS is limited to providing percent energy at the national-level and not by state. Fourth, the analysis is descriptive in nature, and we did not account for confounding factors. However, applying other available methodologies should help to identify potential confounders in future research [18].

Lastly, we were unable to examine the specific food categories contributing to added sugars. However, similar to our study, another nationally representative sample found that nearly 70% of added sugars intake comes from five food categories: sweetened beverages, desserts and sweet snacks, coffee and tea, candy and sugars (e.g., jams, syrups), and breakfast cereals/bars [6]. We expect that the sources/contributors are likely similar, though there might be regional differences in frequency of consumption, accounting for differences in total added sugars consumption by state.

For many, it is important to reduce empty calories and the consumption of added sugars because they hinder the ability to accommodate healthy dietary patterns without exceeding energy needs [5]. For example, substituting water for SSB may improve metabolic health amongst adults with obesity [19]. Our findings may inform state and national efforts to reach the Healthy People 2030 goals [20] to reduce added sugars intake to support health.

So What?

What is already known about this topic?

Excess intake of dietary-added sugars is associated with adverse health consequences.

What does this article add?

This is the first study to report added sugars intake for all 50 states and DC. Overall, US adults consume about 17 tsp/day (68 g), ranging from 14.8 tsp (59.2 g)/day in Alaska to 21.2 tsp (84.8 g)/day in Kentucky in 2010 and 2015. Added sugars intake varied by state and sociodemographic characteristics, which may contribute to chronic disease disparities.

What are the implications for health promotion practice or research?

Our findings may inform state and national efforts to reduce added sugars intake to support optimal health.

## Figures and Tables

**Table 1:** Estimated mean dietary-added sugars intake (teaspoon/day) among US adults aged 18 or older (N = 52,279), United States, 2010 and 2015 ^a^.

Characteristic	No. Respondents (Unweighted)	Estimated Dietary Added Sugars Intake (tsp/Day), Weighted Mean ± Standard Error	*p*-Value
Total	52,279	17.0 ± 0.1	
Age, years			
18–24	5011	21.3 ± 0.2 *	<0.001
25–39	13,913	19.1 ± 0.1 *	<0.001
40–59	17,727	16.3 ± 0.1 *	<0.001
≥60	15,628	13.5 ± 0.1	Reference
Sex			
Male	23,348	19.7 ± 0.1 *	<0.001
Female	28,931	14.4 ± 0.1	Reference
Race/ethnicity			
White, non-Hispanic	31,337	16.8 ± 0.1	Reference
Black, non-Hispanic	7499	17.9 ± 0.2 *	<0.001
Hispanic	9235	17.9 ± 0.1 *	<0.001
Other, non-Hispanic	4208	15.5 ± 0.2 *	<0.001
Marital status			
Married/domestic partnership	26,272	16.7 ± 0.1 *	Reference
Not married	26,007	17.5 ± 0.1 *	<0.001
Education			
<High school	7967	18.9 ± 0.2 *	<0.001
High school/GED	13,286	18.4 ± 0.1 *	<0.001
Some college	16,087	17.1 ± 0.1 *	<0.001
College graduate	14,939	14.9 ± 0.1	Reference
Annual household income			
<$35,000	21,756	18.0 ± 0.1 *	<0.001
$35,000–$74,999	15,939	17.2 ± 0.1 *	<0.001
$75,000–$99,999	5386	16.7 ± 0.2 *	<0.001
≥$100,000	9198	15.6 ± 0.1	Reference
Metropolitan/nonmetropolitan status ^b^			
Metropolitan	43,243	16.8 ± 0.1	Reference
Nonmetropolitan	9036	18.5 ± 0.2 *	<0.001
Census region ^c^			
Northeast	8376	16.5 ± 0.2 *	<0.01
Midwest	11,288	17.3 ± 0.1 *	<0.001
South	18,513	17.8 ± 0.1 *	<0.001
West	14,102	16.0 ± 0.1	Reference

* Significant difference in the mean estimated added sugars intake (tsp/day) using a *t*-test at the *p* < 0.05 level. ^a^ Data are for 50 states and the District of Columbia. Estimated dietary-added sugars intake was calculated based on respondents’ answers to nine questions: During the past month, how often did you (1) “… drink regular soda or pop that contains sugars? Do not include diet soda?”; (2) “… drink SPORTS and ENERGY drinks such as Gatorade, Red Bull, and Vitamin water?”; (3) “… drink sweetened fruit drinks, such as Kool-Aid, cranberry and lemonade? Include fruit drinks you made at home and added sugars to.”; (4) “…, drink coffee or tea that had sugars or honey added to it? Include coffee and tea you sweetened yourself and presweetened tea and coffee drinks such as Arizona Iced Tea and Frappuccino. Do not include artificially sweetened coffee or diet tea.”; (5) “… eat chocolate or any other types of candy? Do not include sugars-free candy.”; (6) “… eat doughnuts, sweet rolls, Danishes, muffins or pop-tarts? Do not include sugars-free items.”; (7) “… eat cookies, cake, pie or brownies? Do not include sugars-free kinds.”; (8) “… eat ice cream or other frozen desserts? Do not include sugars-free kinds.”; (9) “… eat hot or cold cereals?”. ^b^ Based on National Center for Health Statistics Urban–Rural Classification Scheme for Counties (https://www.cdc.gov/nchs/data_access/urban_rural.htm) (Accessed on 8 December 2022). Metropolitan includes large central metro, large fringe metro, medium metro, and small metro categories. Nonmetropolitan includes micropolitan and noncore categories. ^c^ US Census Bureau-defined regions: Northeast (Connecticut, Maine, Massachusetts, New Hampshire, New Jersey, New York, Pennsylvania, Rhode Island, Vermont); Midwest (Illinois, Indiana, Iowa, Kansas, Michigan, Minnesota, Missouri, Nebraska, North Dakota, Ohio, South Dakota, Wisconsin); Southern (Alabama, Arkansas; Delaware, District of Columbia, Florida, Georgia, Kentucky, Louisiana, Maryland, Mississippi, North Carolina, Oklahoma, South Carolina, Tennessee, Texas, Virginia, West Virginia); and Western (Alaska, Arizona, Colorado, Hawaii, Idaho, Montana, Nevada, New Mexico, Oregon, Utah, Washington, Wyoming).

**Table 2:** Estimated mean dietary-added sugars intake (teaspoon/day) among US adults aged 18 or older (N = 52,279), United States, 2010 and 2015 ^a^.

Census Region, State	No. Respondents	Estimated Dietary Added Sugars Intake (tsp/Day), Mean ± Standard Error
Northeast		
Pennsylvania	2467	18.0 ± 0.3
New Hampshire	501	17.0 ± 1.6
Connecticut	596	16.5 ± 0.6
Maine	607	16.1 ± 0.5
Rhode Island	361	16.1 ± 0.7
Vermont	342	16.0 ± 1.0
Massachusetts	787	15.9 ± 0.4
New York	1103	15.9 ± 0.3
New Jersey	1103	15.8 ± 0.3
Midwest		
South Dakota	507	18.7 ± 1.6
Indiana	956	18.1 ± 0.4
Michigan	1347	17.9 ± 0.4
Kansas	778	17.8 ± 0.6
Iowa	703	17.7 ± 0.7
Ohio	1554	17.6 ± 0.4
Missouri	819	17.0 ± 0.5
Illinois	1812	16.9 ± 0.4
Nebraska	577	16.8 ± 0.4
North Dakota	478	16.6 ± 0.3
Minnesota	955	16.2 ± 0.4
Wisconsin	802	16.1 ± 0.3
South		
Kentucky	841	21.2 ± 0.6
Mississippi	623	20.0 ± 0.9
Oklahoma	630	19.8 ± 0.9
Arkansas	565	19.3 ± 0.6
West Virginia	528	19.1 ± 0.9
Alabama	760	18.7 ± 0.5
Tennessee	839	18.5 ± 0.3
South Carolina	682	18.4 ± 0.7
Georgia	1449	18.1 ± 0.4
North Carolina	1391	17.8 ± 0.7
District of Columbia	511	17.6 ± 0.7
Louisiana	702	17.6 ± 0.6
Texas	3922	17.5 ± 0.2
Maryland	757	16.9 ± 0.5
Virginia	1010	16.7 ± 0.4
Delaware	409	16.2 ± 0.7
Florida	2894	16.2 ± 0.3
West		
Hawaii	494	18.3 ± 0.4
Arizona	844	17.3 ± 0.5
Utah	710	17.1 ± 0.5
New Mexico	674	17.0 ± 0.5
Colorado	834	16.8 ± 0.4
Wyoming	473	16.7 ± 0.3
Nevada	577	16.4 ± 0.5
California	6166	15.7 ± 0.2
Idaho	507	15.6 ± 0.9
Montana	447	15.4 ± 0.6
Washington	1124	15.4 ± 0.3
Oregon	678	15.0 ± 0.4

^a^ Data are for 50 states and the District of Columbia.

## Data Availability

Data is unavailable due to privacy. Use of restricted geocodes (urban/rural residence) required Index data linked to NHIS data at the Research Data Center.
